# 

**Published:** 1894-11

**Authors:** 


### Ohio State Dental Society.

The annual meeting of this Association will be held on Tuesday, December 4th, in Columbus. Though we have not the full programme at hand we are assured the Executive Committee is using every effort to make this a large and interesting meeting. A good programme is well nigh completed.

This society always has for consideration a number of subjects of interest to the entire profession of the State; and in order that these may be properly considered and acted upon, for the largest interest, it is important that, at least, all the better and more representative part of the profession be present.

The question of a Tri-State meeting, which was initiated at the last meeting of this society, has met with a hearty response on the part of Indiana and Michigan, and that meeting to be held in June, 1895, promises to be a very large and important one.

A report of the General Committee, having that matter in charge, will be made at this meeting of the State Society, and there will doubtless be many details to be considered and arranged, for that occasion; and it is very desirable that the profession of Ohio should be made entirely aware of all the arrangements, and the earnest interest and co-operation of every one elicited for that occasion.

There are some other questions of great importance that are, or should come up, for consideration at this meeting.

We hope to meet every representative man of the profession of Ohio in Columbus at this meeting.

### THE DENTAL REGISTER,

**A MONTHLY MAGAZINE DEVOTED TO THE INTERESTS OF THE**

### DENTAL PROFESSION.

*Terms Reduced to* $2.00 *Per Year. Single Copies,* 25 *Cents.*

**EDITED BY PROF. J. TAFT, D.D.S.**

—AND—

**Published by SAM'L A. CROCKER & CO., Ohio Dental and Surgical Depot.**

The volume commences in January and closes in December.

The REGISTER being issued monthly is always fresh, therefore Indispensable to the practitioner who desires to keep posted up with the new inventions and improvements which are daily developed. Neither expense nor effort will be spared to give value and variety to its contents. Articles will be illustrated with well executed engravings whenever they will add to the interest or assist to explanation.

Its extensive circulation and frequency of issue make it one of the BEST DENTAL ADVERTISING MEDIUMS in the United States.

All subscriptions must begin with January or July.

Local or special notices, five lines or less, will be inserted for $3 each insertion; over five lines, 50 cts. per line.

All advertising bills payable in advance, or at furthest, after the issue of the first number containing the advertisement. All transient advertisements to be paid for in advance.

☞ CONTRIBUTIONS SOLICITED.

Exclusively Editorial Communications should be addressed to the Editor.

The latest number of the REGISTER may be ordered with or without other goods any time, and all letters should be addressed to

